# Safety of implantable Collamer lens implantation without ophthalmic viscosurgical device: A retrospective cohort study

**DOI:** 10.1097/MD.0000000000020691

**Published:** 2020-06-12

**Authors:** Manqiang Peng, Qiongyan Tang, Libei Zhao, Muhanmad Ahmad Khan, Ding Lin

**Affiliations:** a Central South University Aier School of Ophthalmology; b Changsha Aier Eye Hospital, Changsha, China.

**Keywords:** balanced salt solution, implantable Collamer lens, ophthalmic viscosurgical device, safety

## Abstract

To compare the safety of implantable Collamer lens (ICL) implantation with and without ophthalmic viscosurgical device (OVD).

A total of 148 eyes underwent a conventional ICL implantation with OVD (OVD group), and 112 eyes underwent a modified ICL implantation without OVD (OVD-free group). The balanced salt solution was used to load ICL and maintain the anterior chamber in the OVD-free group. The surgical time, postoperative uncorrected distance visual acuity, intraocular pressure, endothelial cell density (ECD), and percentage of hexagonal cells were compared between the OVD and the OVD-free groups.

No significant differences were detected in uncorrected distance visual acuity, intraocular pressure, ECD, and percentage of hexagonal cells at any time post-surgery between the 2 groups (*P* > .05). The mean ECD loss was 1.9% in the OVD-free group and 2.3% in the OVD group at 2 years post-surgery (*P* = .680). The surgical time was much shorter in the OVD-free group than that in the OVD group (*P* ≤ .001). None of the following occurred at any time during the 2-year follow-up period in both groups: cataract formation, macular degeneration, or any other vision-threatening complications.

OVD-free ICL implantation presented satisfactory results for safety. Compared to OVD, the OVD-free technique had the advantages of decreased surgical time, increased efficiency, and reduced cost.

## 1 Introduction

The Visian implantable Collamer lens (ICL) (Staar Surgical AG, Nidau, Switzerland) has shown excellent outcomes for the correction of ametropias.^[1–8]^ However, several postoperative complications, including crystalline lens opacities, increased intraocular pressure (IOP) and pupillary block, and endothelial cell loss, have been reported. An early rise in IOP was reported to be relatively frequent.^[9]^ An ophthalmic viscosurgical device (OVD) left in the eye induces a postoperative IOP spike.^[10]^ Senthil et al^[11]^ reported a single case wherein the central part of the ICL was blocked with viscoelastic and inflammatory debris. Thus, OVD must be thoroughly removed from the anterior chamber after usage, especially from the space between the ICL and anterior capsule. However, it is a surgical challenge to remove the OVD completely from behind the ICL. Interestingly, the novel model V4c Visian ICL has been designed with a central hole of 0.36 mm to reduce the incidence of cataracts.^[12]^ Nevertheless, the cannula irrigation with enforced stream onto the capsule through that hole during removal of OVD may induce anterior subcapsular cataract (ASC).^[13]^

In order to avoid these complications, we used balanced salt solution (BSS) since March 2016 instead of OVD to complete the operation. The present retrospective study aimed to assess the safety of this technique.

## 2 Methods

### 2.1 Patients

This retrospective study included 260 eyes of 194 patients, who underwent implantation of a V4c ICL or Toric ICL to correct myopia, from March 2016 to October 2018. Of these, 148 eyes underwent a conventional ICL implantation with OVD, and 112 eyes underwent a modified ICL implantation without OVD. The study protocol was approved by the Ethics Committee of Changsha Aier Eye Hospital, Changsha, China. All patients were fully informed of the details and potential risks of the procedure, and written informed consent was obtained from all patients. The study followed the tenets of the Declaration of Helsinki.

The inclusion criteria were stable refraction for 2 years and clear central cornea before the surgery. The exclusion criteria were for the eyes were as follows: anterior chamber depth (ACD) <2.8 mm, cataract, history of glaucoma or retinal detachment, macular degeneration, or retinopathy, neuro-ophthalmic diseases, and history of ocular inflammation.

### 2.2 Examinations

Before surgery, all the patients underwent a complete ophthalmological examination. The evaluation target was emmetropia in all cases and included logarithm of the minimal angle of resolution (logMAR) of the uncorrected distance visual acuity (UDVA), logMAR of the corrected distance visual acuity (CDVA), manifested and cycloplegic refractions, keratometry, corneal topography and pachymetry (Pentacam HR, Oculus Optikgeräte GmbH, Wetzlar, Germany), ACD and axial length (IOLMaster, Zeiss Humphrey, Carl Zeiss Meditec, Inc., Dublin, CA), endothelial cell density (ECD) and percentage of hexagonal cells (HEX) (SP-3000P, Topcon Co., Tokyo, Japan), slit-lamp microscopy (SL-1E, Topcon), IOP (Computerized Tonometer CT-80A, Topcon), and binocular indirect ophthalmoscopy through a dilated pupil.

### 2.3 Surgical technique

All surgeries were performed by the same surgeon (DL) under topical anesthesia. On the day of surgery, the patients were administered topical tropicamide (5 mg/mL) and phenylephrine hydrochloride (5 mg/mL) eyedrops for pupil dilation.

**Figure 1:**
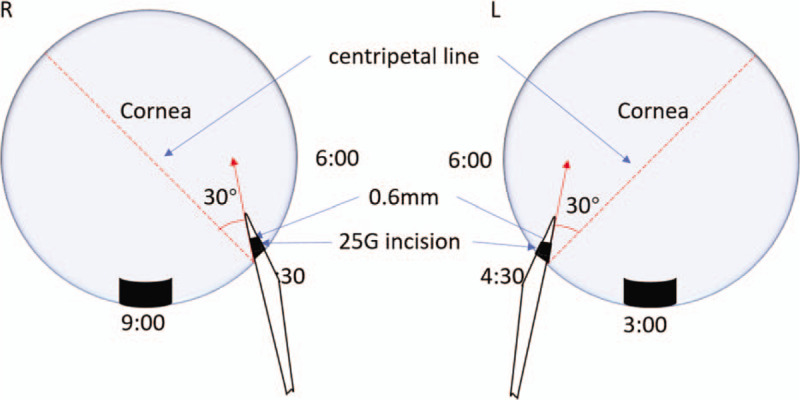
Illustration of the direction of 25 G incision. A 0.6 mm incision at the 7:30 o’clock-position in the right eye or 4:30 o’clock-position in the left eye. The edge of the knife was facing 6:00 o’clock, and puncture was made at an angle of 30° to the centripetal line. The width of the inner incision was about 0.6 mm. The length of the incision was about 1.2 mm.

**Figure 2:**
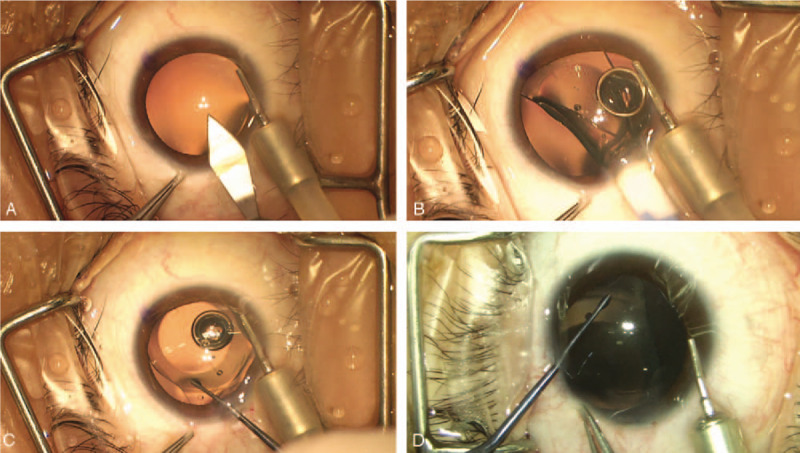
Implanting the implantable Collamer lens. (A) A temporal 3.0 mm clear corneal incision was created. (B) The anterior chamber was well-formed by balanced salt solution. The implantable Collamer lens was gradually injected into the eye. (C) The tip of the haptics was placed behind the iris by a phakic intraocular lens manipulator through the 3 mm incision. (D) In the earlier practice stage, the tip of the haptics was placed through an extra 1.0 mm side-incision.

In the OVD-free group, the ICL was loaded with BSS in the cartridge and tip (Staar Surgical Co.). An incision made by a microsurgical 15° stab knife (Sharpoint, Surgical Specialties Corp.: Reading, PA, USA) for 25-gauge (G) infusion cannula at the 7:30 o’clock positions in the right eye or 4:30 o’clock positions in the left eye. Next, we designed the standard direction and length of the incision to match the 25 G infusion. The edge of the knife was facing 6:00 o’clock, and puncture was made at an angle of 30° to the centripetal line. The width of the inner incision was about 0.6 mm, which was slightly larger than the size of 25 G infusion cannula (the outer diameter of the cannula was 0.56 mm)m, and the length of the incision was approximately 1.2 mm (Fig. 1). The anterior chamber was fully filled with BSS after inserting the 25 G infusion cannula connected to the BSS infusion media bottle. The pressure of the infusion was controlled by the BSS bottle, which was 60 to 80 cm higher than the patient's eye. Then, a temporal 3.0 mm clear corneal incision was created (Fig. 2A), and the ICL was injected into the anterior chamber through this 3 mm incision (Fig. 2B). Subsequently, a phakic intraocular lens manipulator was introduced through the incision to place the tip of the haptics behind the iris (Fig. 2C). In the earlier practice stage, we made an extra 1.0 mm side-incision to place the tip of the haptics (Fig. 2D). After situating the ICL into the ciliary sulcus, the 25 G infusion cannula was removed. The accuracy of the ICL position was verified before the intraocular miotic agents were administered to decrease the pupil size.

In the OVD group, the ICL was loaded with OVD and injected into the anterior chamber, which was fully filled with OVD through the 3 mm incision and the haptics were placed through the side incision.

The time of surgery and any eventual complications during implantation were noted during the operation.

### 2.4 Follow-up

Postoperative examinations were conducted at 2 hours, 1 day, 1 week, 1 month, 3 months, 6 months, 1 year, and 2 years after the surgery. All patients were advised to contact the hospital for an extra visit any time if they feared a potential complication. The complications were analyzed as a measure of the safety of the procedure.

### 2.5 Data analysis

Independent Student *t* test was used to compare the age, axial length, ACD, surgical time, UDVA, CDVA, safety index, IOP, ECD, and percentage of HEX between the 2 groups. Paired Student *t* test was used to compare the preoperative and postoperative IOP. A normal distribution check (Kolmogorov–Smirnov test) was performed to validate the use of the Student *t* test. Comparison of gender and ICL type (ICL/Toric ICL) were analyzed using the Chi-square test. *P*-value < .05 was considered statistically significant. All data were analyzed using SPSS software (version 22.0, IBM Corp., Armonk, NY).

## 3 Results

The demographic data are shown in Table 1. No statistically significant differences were detected in age, gender, axial length, ICL type, UDVA, CDVA, IOP, ACD, ECD, and percentage of HEX between the 2 groups before the surgery.

**Table 1:**
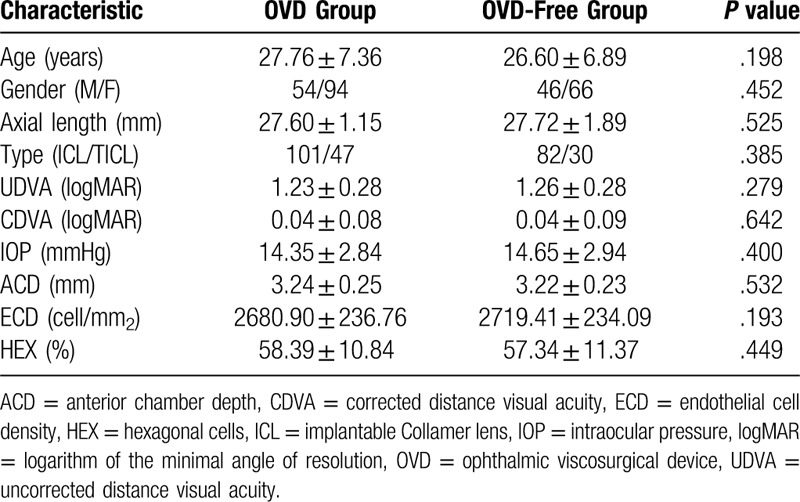
Preoperative patient demographics.

**Figure 3:**
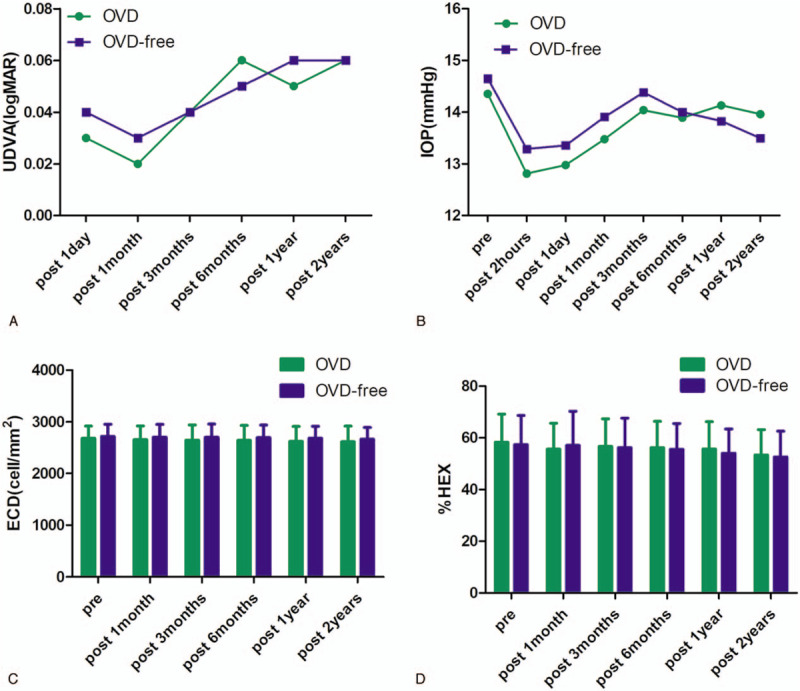
Time course of postoperative uncorrected distance visual acuity (A), intraocular pressure (B), endothelial cell density (C), and percentage of hexagonal cells (D) after implantable Collamer lens implantation in the OVD group and OVD-free group. OVD = ophthalmic viscosurgical device.

The surgery time was calculated from the beginning of the load of ICL to the end of the removal of eye speculum. The mean surgical time was much shorter in the OVD-free group (189.38 ± 29.14 seconds) than that in the OVD group (318.99 ± 38.55 seconds) (*P* ≤ .001). In addition, no statistically significant difference was observed in postoperative UDVA, IOP, ECD, and percentage of HEX between the 2 groups at any time (Fig. 3, *P* > .05). The IOP decreased significantly at 2 hours, 1 day, and 1 month in the OVD group and 2 hours, 1 day, 1 month, 6 months, 1 year, and 2 years post-surgery in the OVD-free group (*P* < .05). The postoperative IOP was > 22.0 mm Hg and 5 mm Hg or more above the preoperative IOP in 2 cases (28.3 mm Hg, increased 12.6 mm Hg; 24.3 mm Hg, increased 7.7 mm Hg) at 1 month post-surgery in the OVD group and 2 cases (29.8 mm Hg, increased 18.8 mm Hg; 24.7 mm Hg, increased 9.0 mm Hg) at 1 month post-surgery in the OVD-free group. The safety index is the ratio of postoperative CDVA to preoperative CDVA and was 1.12 in the OVD-free group and 1.11 in the OVD group at 2 years post-surgery (*P* = .520). The mean ECD loss was 1.9% in the OVD-free group and 2.3% in the OVD group at 2 years post-surgery (*P* = .680).

In the OVD-free group, intraoperative complications included sudden collapses of the anterior chamber due to the haptic stuck in the 3 mm incision (1 eye, 0.9%) and contact between the haptics and the peripheral corneal endothelium (3 eyes, 2.7%). No other intraoperative complication was found in either of the groups. Furthermore, none of the following occurred at any time during the 2-year follow-up period in the 2 groups: cataract formation, macular degeneration, or any other vision-threatening complication. An ICL exchange occurred in 1 eye (0.68%) in the OVD group, due to incorrect initial sizing.

## 4 Discussion

Several OVD-free techniques were developed to implant IOL effectively. Harshul et al^[14]^ described a technique called hydroimplantation for the implantation of 1-piece acrylic foldable IOL using an irrigation cannula of the phaco machine without OVD. Approximately 400 cases were treated in 1 year in this study without any posterior capsule rupture or other complications. The hydroimplantation technique has the advantage of increasing efficiency, reducing surgery time and cost, and elevating IOP postoperatively. Zu et al^[15]^ described a technique using Simcoe cannula as an anterior chamber maintainer (ACM) for the implantation of posterior chamber IOLs and even anterior chamber IOLs without OVD. With a vast experience of implanting thousands of IOLs, the authors confirmed that IOL implantation using the Simcoe cannula as an ACM was a safe and cost-effective technique, which was especially useful to surgeons in the developing countries. As the technique of ICL implantation was similar to that of IOL implantation, we applied this technique to ICL implantation in the present study. Compared to the irrigation and Simcoe cannulae, we preferred to use 25 G cannula as an ACM for infusion. The 25 G cannula rarely prolapses from the incision when it is fixed at the incision even if the patients suddenly move their eyes.

In this study, we found that the mean postoperative UDVA was similar and stable during the 2-year follow-up in both groups. The safety index was 1.12 and 1.11 in the OVD-free and OVD group, respectively. Compared to the previous study by Huseynova et al^[16]^ and Lisa et al^[17]^ that reported safety indices of 1.14 and 1.04, respectively, our study showed similar outcomes.

Regarding adverse events, the major concerns included altered IOP, ECD loss, ASC opacity, or visually significant cataract.^[18]^ The IOP was similar (*P* > .05) between the 2 groups at any time. Kazutaka et al^[6]^ noted that the IOP significantly decreased at 1 day and 1 week after ICL implantation. The present research also showed that the IOP decreased significantly at 2 hours, 1 day, and 1 month in the OVD group and 2 hours, 1 day, 1 month, 6 months, 1 year, and 2 years post-surgery in the OVD-free group (*P* < .05). The IOP was >22 mm Hg and increased >5 mm Hg in 4 cases (2 cases in OVD group and 2 cases in OVD-free group) at 1 month post-surgery, which might be due to the effect of postoperative inflammation and topical steroids.^[19]^ After topical steroid and hypertensive treatments for 3 to 10 days, no eye required further hypertensive treatment to maintain IOP.

Interestingly, no significant differences were detected between the 2 groups in ECD and percentage of HEX at any time in this study (*P* > .05). The ECD loss at 2 years post-surgery was lower than the mean endothelial cell loss of 21 studies including data on 1,476 eyes with weighted average follow-up of 14.7 months in a literature review (2.6%, range: 0.1%–9.0%).^[18]^ Neither ASC opacity nor visually significant cataract were observed during the 2-year follow-up in our study. The occurrence of intraoperative complications in this study was low and mainly observed in the earlier practice stage. With increased experience, these complications could be avoided. Additionally, we found that UDVA and central endothelium were not affected in these eyes (UDVA 0.0-0.1 logMAR, ECD loss 1.4%–2.2%) at 2 years post-surgery.

The present study also suggested that the OVD-free technique had the advantage of reduced surgery duration, which might increase the comfort of the patient. Moreover, it can decrease the cost and increase the efficiency of the technique, which were critical factors in high-volume surgical centers in developing countries with a large population.

There were several key points for a successful surgery. First, the infusion pressure was about 60 to 80 cmH_2_O. Lower pressure would lead to instability of the anterior chamber, and higher pressure would lead to increased damage. Second, 15° knife was better than 25 G needle in making the 25 G incision. The 15° knife could be used to make a bigger outer and smaller inner incision, which was conducive to fix the 25 G infusion. The incision made by 25 G needle was slightly smaller, such that the 25 G infusion cannula could not be inserted easily, and the incision was difficult to close. In addition, the 25 G needle was long and could easily injure the lens. Third, accurate direction, width, and length of incision were vital. The appropriate length of incision was 1.0–1.5 mm. The 25 G tube fixation would be difficult when the length of the incision was <1.0 mm, and corneal damage would occur when the length was >1.5 mm. The appropriate width of incision was 0.4–0.8 mm. The tube could easily slip out if the width of the incision was >0.8 mm. However, the tube was hard to be inserted when the width of the incision was <0.4 mm. Incorrect puncture direction would lead to some problems. The ICL implantation was difficult when puncturing in centripetal direction, and cornea or lens would be damaged when puncturing in the horizontal direction. Fourth, placing the tip of the haptics accurately. In the earlier stage, we made an extra 1.0 mm side-incision to place the tip of the haptics. With some experience, we found that the anterior chamber could be stabilized through the 3.0 mm incision due to the continuous infusion.

Nevertheless, the present study has several limitations. First, the OVD-free technique had limitations in compromised anterior chamber stability compared with OVD, especially for placing the haptics. The surgery technique was critical for avoiding intraoperative complications. Second, the study was performed in a retrospective manner. Thus, a prospective randomized study would be ideal for confirming our results. Third, the peripheral corneal endothelium was not checked. Hence, additional cases are needed to confirm the incidence of the intraoperative complications and their effects on the corneal endothelium, especially the peripheral corneal endothelium with the OVD-free technique.

In conclusion, the present study demonstrated that OVD-free ICL implantation was satisfactory with respect to safety. Compared to OVD, the OVD-free technique had the advantages of decreased surgical time, increased efficiency, and reduced cost. Therefore, OVD-free ICL implantation may be an attractive option for experienced surgeons and patients, especially in high-volume surgical centers. However, to confirm its long-time safety, peripheral corneal endothelium, long-term follow-up, and additional cases would be required in future studies.

## Acknowledgments

The authors would like to thank clinical team of the Refractive Department of Changsha Aier Hospital for their contribution of patient arrangements and Zengping Liu for general support in present study.

## Author contributions

**Conceptualization:** Ding Lin.

**Data curation:** Manqiang Peng, Libei Zhao.

**Formal analysis:** Qiongyan Tang, Muhanmad Ahmad Khan.

**Investigation:** Manqiang Peng, Libei Zhao.

**Methodology:** Ding Lin, Qiongyan Tang.

**Resources:** Libei Zhao, Muhanmad Ahmad Khan.

**Supervision:** Qingyan Tang.

**Writing – original draft:** Manqiang Peng.

**Writing – review & editing:** Ding Lin, Qiongyan Tang, Manqiang Peng.
